# Meeting report of the third annual Tri-Service Microbiome Consortium symposium

**DOI:** 10.1186/s40793-020-00359-6

**Published:** 2020-07-13

**Authors:** J. Philip Karl, Robyn A. Barbato, Laurel A. Doherty, Aarti Gautam, Sarah M. Glaven, Robert J. Kokoska, Dagmar Leary, Rebecca L. Mickol, Matthew A. Perisin, Andrew J. Hoisington, Edward J. Van Opstal, Vanessa Varaljay, Nancy Kelley-Loughnane, Camilla A. Mauzy, Michael S. Goodson, Jason W. Soares

**Affiliations:** 1 Military Nutrition Division, United States Army Research Institute of Environmental Medicine, Natick, MA USA; 2 United States Army Cold Regions Research and Engineering Laboratory, Hanover, NH USA; 3 Soldier Performance Optimization Directorate, United States Army Combat Capabilities Development Command Soldier Center, Natick, MA USA; 4 Medical Readiness Systems Biology, Center for Military Psychiatry and Neuroscience, Walter Reed Army Institute of Research, Silver Spring, MD USA; 5 Center for Bio/Molecular Science and Engineering, Naval Research Laboratory, Washington, DC USA; 6 Physical Sciences Directorate, United States Army Research Laboratory – United States Army Research Office, Research Triangle Park, Durham, NC USA; 7 Center for Biomolecular Science & Engineering, United States Naval Research Laboratory, Washington, DC USA; 8 American Society for Engineering Education, Washington, DC USA; 9 Biotechnology Branch, United States Army Combat Capabilities Development Command-Army Research Laboratory, Adelphi, MD USA; 10 Department of Systems Engineering and Management, Air Force Institute of Technology, Wright-Patterson AFB, Dayton, OH USA; 11 Military and Veteran Microbiome: Consortium for Research and Education, Aurora, CO USA; 12 Veterans Health Administration, Rocky Mountain Mental Illness Research Education and Clinical Center, Rocky Mountain Regional Veterans Affairs Medical Center, Aurora, CO USA; 13 Department of Physical Medicine & Rehabilitation and Center for Neuroscience, University of Colorado Anschutz Medical Campus, Aurora, CO USA; 14 Human Systems Directorate, Office of the Underscretary of Defense for Research & Engineering, Washington, DC USA; 15 Soft Matter Materials Branch, Materials and Manufacturing Directorate, Air Force Research Laboratory, Wright-Patterson AFB, Dayton, OH USA; 16 711th Human Performance Wing, Air Force Research Laboratory, Wright-Patterson AFB, Dayton, OH USA

**Keywords:** Microbiota, Environmental microbiome, Military, Human performance, Microbiome engineering, Polymicrobial communities, Biotechnology, Synthetic biology

## Abstract

The Tri-Service Microbiome Consortium (TSMC) was founded to enhance collaboration, coordination, and communication of microbiome research among U.S. Department of Defense (DoD) organizations and to facilitate resource, material and information sharing among consortium members. The 2019 annual symposium was held 22–24 October 2019 at Wright-Patterson Air Force Base in Dayton, OH. Presentations and discussions centered on microbiome-related topics within five broad thematic areas: 1) human microbiomes; 2) transitioning products into Warfighter solutions; 3) environmental microbiomes; 4) engineering microbiomes; and 5) microbiome simulation and characterization. Collectively, the symposium provided an update on the scope of current DoD microbiome research efforts, highlighted innovative research being done in academia and industry that can be leveraged by the DoD, and fostered collaborative opportunities. This report summarizes the presentations and outcomes of the 3rd annual TSMC symposium.

## Introduction

The Tri-Service Microbiome Consortium (TSMC) was founded in 2016 to enhance collaboration, coordination, and communication of microbiome research among U.S. Department of Defense (DoD) organizations and to facilitate resource, material and information sharing among consortium members. Towards those goals, and to discuss applications and implications of microbiome research, the TSMC hosts an annual symposium that includes subject matter experts from federal/state agencies, DoD-affiliates, academic institutions, and industry [1–3]. The 2019 symposium was held 22–24 October at Wright-Patterson Air Force Base in Dayton, OH. Over the 3 day symposium, 45 speakers (51% female, 13% underrepresented minority) which included 26 speakers (10 early-career) from government laboratories (Table 1), four from DoD-affiliated laboratories, twelve from academic institutions, and three from commercial companies presented on microbiome-related topics within five broad thematic areas: 1) human microbiomes; 2) transitioning products into Warfighter solutions; 3) environmental microbiomes; 4) engineering microbiomes; and 5) microbiome simulation and characterization. Additionally, 130 registered attendees (65% government, 8% DoD-affiliated, 13% academia, 14% industry) engaged with 30 poster presenters (83% government, 7% DoD-affiliated and 10% industry), and participated in mediated discussions centered on bioinformatics and resource sharing. This report details those activities in an effort to foster potential collaboration across scientific communities.

**Table 1:** Summary of presented DoD microbiome-related research

Organization	Thematic area	Topic
*Army*		
CCDC SC/ USARIEM	Human microbiomes: Warfighter performance (stress, diet and the gut microbiome)	Use of in vitro human gut models to extend insight into clinical study on effects of military food rations on the gut microbiota [4, 5]
USACEHR/ WRAIR	Human microbiomes: Warfighter performance (stress, diet and the gut microbiome)	Effects of post-traumatic stress disorder-like stress and microgravity on the microbiome-gut-brain axis in animal models
USUHS	Human microbiomes: Warfighter performance (stress, diet and the gut microbiome)	Gut microbiota response to traveler’s diarrhea and antibiotic treatment
USUHS	Human microbiomes: Warfighter protection (respiratory, nasal, oral and otic microbiomes)	Relations between the nasal microbiota and skin and soft tissue infections in Army recruits [6, 7]
ERDC-EL	Environmental microbiomes (soil and marine)	Effectively using microbes to degrade munitions [8]
ERDC-CRREL	Environmental microbiomes (soil and marine)	Understanding microbial communities during extreme weather events [9]
CCDC-ARL	Environmental microbiomes (polymicrobial communities)	Designing functional microbial consortia for expedient manufacturing [10]
CCDC SC	Emerging tools	Organoid models for studying host-microbe interactions [11]
CCDC SC	Enabling techniques	Increasing physiologic relevance of in vitro gut fermentation models
WRAIR	Enabling techniques	Applications of single cell RNAseq in cellular immunology
*Air Force*		
59th Medical AF	Human microbiomes: Warfighter protection (respiratory, nasal, oral and otic microbiomes)	Temporal shifts in the skin microbiome of Air Force recruits during initial military training [12]
AFIT	Environmental microbiomes (built environment)	Methodological considerations for studying the microbiome of the built environment [13]
AFRL	Polymicrobial communities	Aircraft microbiomes and relation to biocorrosion and biodeterioration [14]
AFRL	Engineering microbiomes	Engineering microbes to sense and respond to physiologic changes in humans
AFRL	Emerging tools	Gut-brain on a chip microfluidic models to study host-microbe interactions
*Navy*		
NAMRU- Dayton	Human microbiomes: Warfighter performance (stress, diet and the gut microbiome)	Potential applications of probiotics for Warfighter performance
NRL	Environmental microbiomes (soil and marine)	Using marine microbes for electricity production [15]
NRL	Environmental microbiomes (polymicrobial communities)	Microbiomes in ship hull biofouling
NRL	Engineering microbiomes	In situ engineering of autotrophic microbial communities [16]
NRL	Enabling techniques	Multi-omics and bioinformatics for microbiome analyses
*DARPA*	Program update	Ongoing DARPA programs supporting research into using microbes for environmental sensing and reporting, modulating mosquito attractiveness, and nasal-based delivery of neuromodulatory microbes.
*DTRA*	Program update	Ongoing DTRA programs to understand radiation effects on microorganisms
*MVM-CoRE* (non-DoD)	Human microbiomes: Warfighter performance (stress, diet and the human microbiome)	US Veteran Microbiome Project status update [17]

*AFIT* Air Force (AF) Institute of Technology; *AFRL* AF Research Laboratory; *ARL* Army Research Laboratory; *CCDC SC* Combat Capabilities Development Command-Soldier Center; *CFD* Combat Feeding Directorate; *CRREL* Cold Regions Research and Engineering Laboratory; *DARPA* Defense Advanced Research Projects Agency; *DoD* US Department of Defense; *DTRA* Defense Threat Reduction Agency; *EL* Environmental Laboratory; *ERDC* US Army Engineer Research and Development Center; *MVM-CoRE* Military and Veteran Microbiome Consortium for Research and Education; *NAMRU* Naval Medical Research Unit; *NRL* Naval Research Laboratory; *USACEHR* US Army Center for Environmental Health Research; *USAMRIID* US Army Medical Research Institute of Infectious Diseases; *USARIEM* US Army Research Institute of Environmental Medicine; *USUHS* Uniformed Services University of the Health Sciences; *WRAIR* Walter Reed Army Institute of Research

### Opening remarks

Opening remarks by TSMC chair, Mr. Jason Soares, Combat Capabilities Development Command Soldier Center, and vice-chair, Dr. Michael Goodson, Air Force Research Laboratory, noted the TSMC’s positioning under the broad umbrella of DoD biotechnology research. The implications of that positioning were highlighted by Dr. Linda Chrisey, Office of the Under Secretary of Defense for Research and Engineering and Office of Naval Research, who provided a keynote address centered on initial steps being taken towards the development of a DoD Biotechnology Modernization Roadmap. Biotechnology was described as a “disruptive technology,” meaning that related research and development efforts could provide distinct capabilities for many aspects of defense and national security. That potential was highlighted as underpinning a need for investment in programs aimed at modernizing DoD research and development to establish the DoD as a global leader in biotechnology. Principle applications of those modernization programs are envisioned to include optimizing and/or enhancing warfighting systems, Warfighter health and performance, military medicine, and chemical biological defense. Examples of applications for microbiology within those areas included rapid vaccine development, smart fabric design, creating infrastructure materials, and advancing biomonitoring tools. Emphasis was placed on the need for the DoD to align with and leverage academia and industry to accomplish biotechnology modernization. The establishment of DoD manufacturing and biotechnology innovation institutes were highlighted as examples of one approach to innovating and advancing biotechnology for health and performance applications within both the commercial space and the DoD. Taken together, these opening remarks framed the importance of DoD microbiome research within the broad context of developing innovative biotechnologies for maintaining and advancing multiple and diverse national security interests.

### Human microbiomes

#### Warfighter performance: stress, diet and the gut microbiome

Initial presentations in the human microbiomes session focused on health conditions prevalent in military personnel, namely traveler’s diarrhea, mild traumatic brain injury, and post-traumatic stress disorder, and their potential relation to the gut microbiome. Dr. Ryan Johnson, Uniformed Services University of the Health Sciences, reported a secondary analysis of data obtained from the Trial Evaluating Ambulatory Therapy of Travelers’ Diarrhea Study [18], concluding that diarrhea severity and geographic location were stronger predictors of post-traveler’s diarrhea gut microbiota composition than was the antibiotic used in treatment. Findings undescored the importance of environmental exposures in restoring a healthy gut microbiome following perturbation. Focusing on the gut-brain axis, Dr. Rasha Hammamieh, Walter Reed Army Institute of Research, described recent studies demonstrating persistent changes in the murine gut microbiome following exposure to post-traumatic stress disorder-like stress [19], and associations between gut microbial metabolites and pain networks in the brain. Together, the results were described as suggesting that bidirectional communication along the microbiota-gut-brain axis may comprise one potential factor influencing the development and progression of post-traumatic stress disorder and pain perception. Dr. Lisa Brenner, Military and Veteran Microbiome Consortium for Research and Education, extended the microbiota-gut-brain axis discussion, describing the Military and Veteran Microbiome Consortium’s ongoing efforts to determine relationships between the gut microbiota and clinical symptomology in individuals with mild traumatic brain injury and post-traumatic stress disorder. Those efforts included establishing the United States Veteran Microbiome Project, which is an ongoing project aiming to serially collect microbiome and health-related data from thousands of Veterans [17].

Dr. Brenner subsequently transitioned into discussing her team’s interest in the potential of probiotics as a treatment strategy for mild traumatic brain injury and post-traumatic stress disorder. She noted that although animal studies provide evidence of biological plausability, few relevant clinical trials currently exist [20]. Discussion around the use of probiotics to improve military health and performance was continued in a separate session by Dr. Richard Agans, Naval Medical Research Unit-Dayton, who presented findings from a recent literature review conducted by a team of TSMC members regarding potential applications of “performance” probiotics in military personnel [21]. Dr. Agans concluded his talk by explaining that although the review found no compelling current evidence to support using any particular probiotic(s) product to support cognitive, physical or psychological performance in healthy military personnel, biological plausibility exists and additional research in military cohorts is needed. In contrast to the discussion on performance probiotics, Dr. Mary Ellen Sanders, International Scientific Association for Probiotics and Prebiotics, focused on potential impacts of probiotics on clinical outcomes. She highlighted a growing evidence base suggesting beneficial effects of probiotics on conditions including antibiotic-associated diarrhea, upper respiratory tract infections, and some digestive disorders [22]. Dr. Sanders opined that traveler’s diarrhea, stress, anxiety, and digestive issues are areas where probiotics may have the greatest likelihood to benefit military personnel, and echoed the sentiment that probiotic studies in military populations are needed. She closed by noting that multiple considerations and challenges will need to be considered in those studies to include probiotic selection (e.g., mechanism of action, strain-specificity and high prevalence of non-responders) and mode of delivery (e.g., shelf-stability, production capacity, dose and matrix).

Additional presentations in the session focused on the importance of nutrient-gut microbiome interactions for health. Dr. Lawrence David, Duke University, and Dr. Eric Martens, University of Michigan, both highlighted published and ongoing work demonstrating the critical importance of dietary fibers for maintaining gut microbiome homeostasis and gut health to include the gut-damaging effects of fiber deficiency [23] and the gut health-promoting effects of prebiotic fibers such as inulin [24]. Finally, Dr. Ida Pantoja-Feliciano, Combat Capabilities Development Command Soldier Center, described a study examining the effects of the US Armed Services Meal, Ready-to-Eat ration on gut microbiota composition and gut health [4]. That work suggested that previous observations of increased intestinal permeability and inflammation in Soldiers consuming military rations in austere environments [25, 26] were more likely due to environmental stressors [27] than any impact of the ration diet on the gut microbiota. Additionally, differential changes in gut microbiota community composition and genetic content following consumption of the ration diet were discovered using an in vitro gut model [5], findings which highlighted how in vitro gut models can be used to study critical nutrient-gut microbiota interactions that cannot be readily examined in vivo.

#### Warfighter protection: skin microbiomes

Skin microbiomes represent a first line of defense between the external world and human body. That point was used in opening remarks by Dr. David Karig, Clemson University, who began the skin microbiome session by describing published and preliminary results from his Army Research Office-funded Multidisciplinary University Research Initiative program focused on characterizing the human skin microbiome. That discussion included an update on his team’s development of bioinformatic tools and multi-scalar analyses of various skin microbiome samples, improvements to metagenomic classifiers [28–31] and visualization tools [32], and documentation of a comprehensive microbial trait database [33]. Application of those tools and resources allowed Karig’s team to demonstrate significant intra-site and inter-personal variation in human skin microbiomes, which will have implications for defining sampling strategies in future studies. Dr. Karsten Zengler, University of California-San Diego, then described his approaches to unravel and predict the network of interactions that drive complex microbial communities, including those of the skin microbiome. Application of those approaches, which included reliance on genome-scale metabolic models [34], demonstrated that the dynamics affecting networking among complex microbial communities depend greatly on specific environmental conditions, the starting species ratios, and individual strain differences [35].

Two presentations discussed efforts to link skin microbiome composition to skin diseases observed among military cohorts. Maj. Thomas Beachofsky, 59th Medical Air Force, presented research that aimed to link changes in clinical skin disease prevalence with changes in the cutaneous microbiomes that result from Air Force trainee communal living and Group A *Streptococcus* prophylaxsis. Among 500 participants, small changes in the skin microbiome were observed over time with a notable inverse relationship between *Staphylococcus* (increased over time) and *Propionibacterium* (decreased over time) [12]. Dr. Scott Merrell, Uniformed University of the Health Sciences, then presented research exploring variation in bacterial composition of different body sites in Army recruits with and without skin and soft tissue infections. Study findings confirmed previous observations that nasal colonization with *Staphyloccocus aureus* positively correlates with skin and soft tissue infections, suggesting that the nasal microbiome may be a reservoir contributing to infection [6, 7]. Both Maj. Beachofsky and Dr. Merrell stressed the need to conduct longitudinal studies to determine whether other microbiomes to which trainees are exposed (e.g., other body sites or the built environment) may be reservoirs of microbes that cause skin disease and infection.

#### Warfighter protection: respiratory, nasal, oral and otic microbiomes

The final session in the human microbiome thematic area delved into less commonly studied human microbiomes. Dr. Gemma Reguera, Michigan State University, discussed the microbiome of the human inner ear and the community’s connection to lung and oropharynx microbiomes. She noted that the closer proximity of the oropharynx to the middle ear than the lung facilitates migration of microbes between the oropharynx and middle ear, and hence similarities in community composition, as would be predicted by ecological theory. She then described differences in the otic microbiomes of divers and non-divers, and ongoing research aiming to develop bacterial replacement therapies for barotrauma. Dr. Robert Dickson, University of Michigan, followed by providing an overview of the human lung microbiome [36]. He described the community as detectable, variable across individuals, transient in health but resident in disease, and predictive of clinical outcomes. These characteristics, it was concluded, highlighted a potential role of the lung microbiome in disease and health warranting further investigation [37].

In summary, presentations within the human microbiomes thematic session highlighted the potential for environmental exposures, stress, nutrients, diets, and dietary supplements to impact human microbiome-host interactions in ways that could either compromise or improve military health and performance. In some instances, those interactions may be influenced by the inter-individual variability in microbiomes described across body sites. A common theme was a need for investment into research conducted in military populations and military environments to ultimately elucidate how human microbiomes may be leveraged for performance optimization and protection.

### Transitioning products into warfighter solutions

Developing human microbiome-targeted solutions for improving Warfighter performance and protection ultimately requires transitioning research into materiel solutions. This session provided insight into challenges and considerations related to developing and transitioning human-microbiome targeted solutions to warfighters. Dr. Sebastin Guery and Ms. Johanna Maukonen, DuPont, Inc., opened the session by noting the complexity of the human gut microbiome, the resulting difficulty that complexity poses for developing personalized solutions to improve human health and cognition, and the long timeline to transitioning basic science to consumer products. Despite such obstacles, they noted promising probiotic and prebiotic interventions exist, and described an extensive microbial library maintained by Dupont, Inc. that provides an emerging capability for probiotic development. Dr. Scott Jackson, National Institutes of Standards and Technology, focused on challenges in the field related to measurement standardization that need to be overcome to advance and transition microbiome research. He discussed a 2019 workshop centered on standards for microbiome measurements, the critical need for microbiome reference materials, and the National Institutes for Standards and Technology’s and other’s efforts to address those challenges. Dr. Jackson emphasized that a major challenge for standardization efforts is making a reference that works for the vast number of different samples, DNA extraction kits, and bioinformatic tools that are used in the field. The final talks focused on regulatory considerations. Dr. Deborah Taylor, Air Force Research Laboratory, discussed lessons learned within DoD microbiome and synthetic biology research programs. She emphasized that because synthetic biology products will be used by warfighters, the regulatory framework needs to stay up-to-date on technological developments to maintain positive public perception and to be proactive in risk assessment. She also stressed the need for ethical considerations and evaluation of risks to be discussed throughout the research and development process. Finally, MAJ Jonathan Stallings, US Army Medical Research and Development Command, closed the session by discussing the Office of Regulated Activities efforts to support scientists within the DoD. He explained that those efforts guide products through regulatory frameworks for commercialization, and echoed the need for standards in human microbiome research to facilitate those processes.

Taken together, these presentations and those within the human microbiome session, highlighted the fact that there is a growing interest in developing and commercializing humanmicrobiome targeted products which may include engineered microbes or microbiomes, and which target performance in addition to health outcomes. However, the expedited transition of products to warfighters will require standardization, and keeping regulatory frameworks, ethical considerations and risk assesments up-to-pace with advances in technologies and product development.

### Environmental microbiomes

#### Built environment

A thematic session focused on the microbiome of the built environment (MoBE) was a first for the TSMC meeting. Dr. Erica Hartmann, Northwestern University, began by summarizing current knowledge on the MoBE. She noted that most microbes in the built environment are not human associated. Rather, she used examples of antimicrobial resistant microorganisms, antimicrobial paint, and sampling of the International Space Station to demonstrate that the chemistry of indoor environments is often strongly connected to the microorganisms inhabiting those environments [38–41]. An underlying theme of all this work was Dr. Hartman’s view of the MoBE as “bags of enzymes that respond to physical and chemical stimuli.” Dr. Jiseon Yang, Arizona State University, expanded discussion on space MoBE by describing results of longitudinal studies of biofilm-forming bacteria recovered from the International Space System’s potable water system [42]. Those studies showed that microbial interactions between different species changed based on the year they were recovered from the water system, and that population dynamics and biofilm structures were impacted by silver disinfectant treatment.

Dr. Wendy Goodson, Air Force Research Laboratory, brought the session back into the atmosphere, presenting her research on aircraft MoBE and on fungal biofilms that can degrade polyurethane and other relevant materials in aircraft [14]. Understanding biodeterioration of aircraft coatings was described as critical for risk analysis, prevention, testing, and evaluation, but also has applications in bioremediation, biorecovery and attritable systems. Dr. Goodson explained that strain specificity was important in aircraft biodeterioration, especially in low nutrient conditions. That observation was notably similar to characeristics of biofilms found in the International Space Station as discussed by Dr. Yang. The session returned to *terra firma* with Mr. Graeme Marsh from zBioscience who introduced the concept of probiotics for environmental health. Specifically, Mr. Marsh discussed probiotic cleaning solutions that use proprietary blends of US Food and Drug Administration Generally Recognized as Safe schedule probiotics and Environmental Protection Agency ‘Safer Choice’ compliant delivery surfactants to putatively reduce existing biofilms for infection control, wound care, and general facility hygiene. Finally, Lt. Col. Andrew Hoisington, Air Force Institute of Technology, discussed his research on MoBE in living and work spaces at the U.S. Air Force Academy, focusing primarily on how the MoBE homogenizes with occupants over time and with increased physical contact [13]. Major findings of his work include that the skin microbiota of two individuals who are not romantically involved or related become more similar with time and that human microbes do contribute to the MoBE in some instances. Resulting microbial signatures might be useful for tracking the sources of the MoBE and for predicting occupants. For example, being able to predict the user of a computer desk or mouse based on the microbiomes found on those items.

#### Soil and marine

A session on soil and marine microbiomes took the discussion of environmental microbiomes outdoors. Dr. Holly Moeller, University of California- Santa Barbara and the Institute of Collaborative Biotechnologies, opened the session by explaining that microbes can extend their phenotype and, in turn, ecological niche through association with other microbes and hosts. She described combining experimentation in the laboratory and the field with mathematical modeling to test hypotheses including how fungal diversity gives plant partners flexibility to adapt to heterogeneous environments [43–45]. Dr. Arvind Varsani, Arizona State University, provided the meeting’s only discussion of viruses, which are underexplored members of many microbiomes, by presenting research on viruses found in Antarctica which play a role in penguin disease and ecosystem health [46, 47]. Notably, viruses were described for their positive effects on host health by limiting the growth of fungal pathogens, emphasizing the need to expand microbiome research beyond bacteria and fungi to understand the ecological role of viruses in microbial ecology. Dr. Robyn Barbato, US Army Engineer Research and Development Center-Cold Regions Research and Engineering Laboratory, took the audience north to Arctic environments, describing the role that microbes play in extreme cold environments, especially in the warming Arctic and subarctic. Dr. Barbato stressed the importance of using appropriate sample collection procedures when microbiome samples are being collected under the harsh conditions of extreme cold environments [9]. She explained that such procedures are required to keep samples viable for controlled laboratory studies aiming to understand interactions between plants and microorganisms in extreme cold environments [48] and the emergence of new microorganisms and functions as permafrost thaws [49].

Moving from microbial ecology towards unique processes of environmental microorganisms, Dr. Fiona Crocker, US Army Engineer Research and Development Center-Environmental Laboratory, and Dr. Rebecca Mickol, Navy Research Laboratory, focused on utilizing bacteria to monitor and modulate the environment. Dr. Crocker discussed monitoring environmental contamination using reptile microbiomes [50] and contaminant degradation using microbes. The latter research demonstrated the importance of amendments to stimulate bacterial consumption of contaminants in different environments [8]. Dr. Mickol emphasized bioelectric processes of marine bacteria to store and produce power [15]. She described a comprehensive metagenomic and metatranscriptomic characterization of a reversible microbial battery showing that the most active organism in the community, an unidentified taxa in the family Desulfobulbaceae, was not the most abundant, drawing attention how commonly used DNA-based techniques do not provide information on microbial activity.

#### Polymicrobial communities

The polymicrobial communities session contributed discussions on different applications of microbes and microbiomes for useful purposes, beginning with presentations centered on engineering microbial consortia for waste material degradation. Dr. Michelle O’Malley, University of California- Santa Barbara and the Institute of Collaborative Biotechnologies, opened by describing an approach to leverage anaerobic consortia from biomass-rich environments (e.g., herbivore guts and feces) dominated by fungi and natural partnerships to turn waste into energy and renewable chemicals [51, 52]. In that work, enrichment cultures provided design rules for engineering consortia, and functional redundancy was a key design parameter to promote consortia stability [53]. Biomass conversion was also discussed by Dr. Matt Perisin, Combat Capabilities Development Command-Army Research Laboratory. Dr. Perisin described the use of co-cultures of anaerobes to build functional microbial consortia that convert waste into commodities [10]. He reported that simulations and genome-scale metabolic models predicted that co-cultures and mixed sugars could direct metabolic pathway fluxes for *Clostridium acetobutylicum* that could be verified using experimental data in the laboratory.

Subsequent talks centered on microbiomes of contaminated field sites, aircraft and ship hull biofilms. Dr. Matthew Fields, Montana State University, compared uncontaminated and uranium-contaminated field sites to show that geochemistry and microbial community structures varied significantly over daily time periods. In those studies, the use of non-canonical amino acid tagging for identifying active cells [54] revealed that while abundant populations were viable, they were not necessarily the most active. Dr. Vanessa Varaljay, Air Force Research Laboratory, focused on microbial communities contaminating aircraft, and reported that while community populations can vary across aircraft, they are mostly dominated by a few key taxa. She also described use of the Joint Biological Decontamination System to rapidly and cost-efficiently decontaminate aircraft with severe mold contamination. Similarly, decontamination was noted as an issue for ship hulls by Dr. Angelina Angelova, Navy Research Laboratory. Dr. Angelova explained that improved biofouling coatings are needed to reduce contamination of ship hulls, that those coating could be developed with greater insight into the offending microbial communities, and described the use and challenges of applying metagenomics towards that aim [55]. Results of such efforts revealed that bacterial diversity was related to the age of the hull biofilm. Moreover, metagenomics analyses showed functional differences with young biofilms on highly active vessels, which were enriched in core metabolism, biosynthesis, and xenobiotic biodegradation, and well matured, aged biofilms on sessile vessels, which were enriched in genetic repair and information processing.

Collectively, presentations within the environmental microbiomes sessions emphasized the complexity of polymicrobial communities within different terrestrial and non-terrestrial environments and the many factors that shape them. Across all talks, research efforts to gain insight into the composition, activity and dynamics of those communities were described as critical to being able to harness the immense potential for manipulating environmental microbiomes for specific needs, such as human and equipment health and performance, power generation and bioremediation.

### Engineering microbiomes

The session on engineering microbiomes highlighted the vast power of synthetic biology to engineer microbiomes for biotechnology applications. Dr. Cynthia Collins, Rensselaer Polytechnic Institute, opened by highlighting several reasons to engineer microbiomes: divide labor within the community; reduce metabolic load on individuals; leverage diverse capabilities of different organisms; and the potential for modular plug-and-play applications. She described engineering microbiomes for applications ranging from biofuel production to health, and using approaches such as co-opting existing communication systems and quorum sensing properties to enable control of interspecies communication between *Escherichia coli* and *Bacillus megaterium*. Dr. Igor Stzepourginski, Eligo Biosciences, provided one example of how engineering microbiomes could be used in human health by illustrating the use of phage-like particles to directly deliver engineered probiotics, termed Eligobiotics, to specific areas of the human microbiome, both internally (i.e., intestines) or externally (i.e., skin). One application of this system is the delivery of a CRISPR-Cas system for sequence-specific killing, for instance in a scenario where antibiotics cannot be used, such as for Shiga-toxin producing *E. coli*. Dr. Amy Breedon, Air Force Research Laboratory, continued the theme of microbiome engineering for human performance by describing efforts to develop biosensors. She explained that microbes can be engineered as factories (producing a range of metabolites), diagnostics (i.e., sense and transduce systems), or smart probiotics (for example, sense, transduce, then produce a metabolite in response). However, despite the potential applications and available tools, a major question is how to sense relevant products. That gap was described as a current bottleneck in sensor development in microbial engineering for human performance. Possible solutions include engineering transcription factors, screening two-component systems, or utilizing G-protein coupled receptors. Finally, Dr. Sarah Glaven, Naval Research Laboratory, explained the need to develop cultivation-free genetics for in situ engineering of microbiome communities where individual isolates are not available. Dr. Glaven focused specifically on a biocathode microbiome capable of microbial electrosynthesis. She noted that while the main constituent, *Candidatus tenderia* electrophaga, cannot be isolated in pure culture, cultivation-free genetics, such as transformation with MAGIC vectors [16] enable top-down engineering of the biocathode microbial community in order to exploit the extracellular electron transfer capabilities of the microbiome. Taken together, the talks focused on engineering microbiomes highlighted novel tools and methods which can be used to engineer microbiomes for a variety of applications to leverage the functional capacity of microbes to modulate human physiology, human and equipment health, and the environment.

### Microbiome simulation and characterization

#### Emerging tools

Technological advancements have, and will continue to promote understanding and ability to manipulate and leverage human and environmental microbiomes. Some of those advancements were highlighted in the “emerging tools” session, beginning with Dr. Harris Wang, Columbia University, who described several recently developed methods and tools that enable high-throughput and low-cost analyses for bacterial RNA sequencing [56], examining spatial metagenomics of microbial communities [57], targeted bacterial engineering, and using microbial recorders for studying gene transfer in complex communities [58]. These tools were described as enablers for culturing of low-abundant strains, low-cost analysis and visualization of community dynamics, the development of microbiome biobanks and disease relevant collections, and engineering of organisms for health and performance applications. Other talks in the session focused on in vitro systems and approaches for studying microbiome-host interactions that cannot be studied, or are difficult to study, in vivo. For example, Dr. Nicholas Guido, Massachusetts Institute of Technology-Lincoln Laboratory, described one application of a 3D-printed artificial gut system that utilizes microfluidics and gas permeable membranes. The system is being used in ongoing research aimed at in situ microbiome engineering, for example engineering a *Bacteroides* strain to sense and respond to bile acids. Dr. Tyler Nelson, Air Force Research Laboratory, expanded on the artificial gut concept by describing the development of a microfluidics-based gut-brain axis-on-a-chip model. The model included a co-culture of Caco2-C2BB, enterocytes, and HT-29 MX goblet cells which interfaced with primary microvascular endothelial cell lumen. While providing examples of the model’s utility, Dr. Nelson described promising preliminary results from experiments in which tryptamine produced in the gut compartment of the model by an engineered *E. coli* strain responding to cortisol was able to be detected in the brain compartment of the model. Finally, Dr. Sarah Pearce, Combat Capabilities Development Command Soldier Center, provided an overview of currently available in vitro and ex vivo models for studying host-microbiome interactions [11]. In particular, she focused on the value of intestinal organoid models for gaining mechanistic insight into gut microbe-host interactions. Intestinal organoid models include multiple intestinal cell types, which were described as providing greater physiologic relevance than traditional single-cell culture. She closed by describing initial efforts to further enhance physiologic relevance of the intestinal organoid models through integration with in vitro gut fermentation systems.

#### Enabling techniques

In the enabling techniques session, Mr. Kenneth Racicot, Combat Capabilities Development Command Soldier Center, reiterated and expanded on Dr. Pearce’s talk, delving further into the integration on in vitro gut fermentation and intestinal organoid models. Mr. Racicot, highlighted the Soldier Center’s joint Army automated colon on a bench (jA^2^COB), which is a simulated in vitro large intestine model that can be used to study nutrient-gut microbiota interactions that cannot be readily examined in vivo. The potential impact of those interactions on host physiology can then be investigated in intestinal and organ-on-a-chip models. Dr. Adam Waickman, Walter Reed Army Institute of Research, continued the theme of using in vitro experimentation to gain mechanistic insight into host-microbiome interactions. Focusing on immune function, Dr. Waickman described how single cell RNA sequencing is being applied for single cell identification and gene expression analysis, rapid antibody development, and for identifying host-microorganism interactions. Techniques for enabling study of environmental microbiomes were also discussed. Dr. Hoi-Ying N. Holman, Lawrence Berkeley National Laboratory, described how synchrotron radiation-based Fourier transform infrared microspectroscopy can be used to study microbiomes, highlighting the use of this technique to study the marine microbiome from the Deepwater Horizon Oil Spill and sulfate reducing bacteria. Dr. Dagmar (Dasha) Leary, Naval Research Laboratory, then discussed automation and application of machine learning to data analysis for ongoing proteomics, metabolomics, and bioinformatics efforts within the Navy. Those efforts were described as essential to the study of marine microbiomes, but not without challenges. For example, proteomics is heavily dependent on metagenomics analyses, but, to date, not many metagenomes for marine samples have been elucidated.

Collectively, presentations within the microbiome simulation and characterization sessions highlighted the emergence of powerful tools, techniques, and methods that will provide investigators opportunities to gain mechanistic insight into how microbial communities interact and influence human physiology and the environment. These approaches were predicted to further efforts to move beyond correlation and identify causal pathways by which microbes influence human and environmental health, disease, and performance, and to develop targeted intervention strategies that leverage the microbiome, including directed engineering of microbes and microbial communities.

### Takeaways and conclusions

Three days of compelling research presentations and discussions during the third annual TSMC symposium provided several key takeaways. Foremost, DoD leadership continues to consider microbiome science as one component of biotechnology research and development needed to advance national security interests. As a result, DoD scientists, along with academia and industry partners, are investing resources and effort into investigating how microbiomes can be used to optimize human health and performance, maintain and improve warfighter systems, and monitor and improve environmental health. Those efforts include fundamental research on where microbes are located, how they impact a larger system (animal or environment), and how they change due to natural succession and/or disturbance. There is tremendous interest in applying that knowledge to developing microbiome-associated biotechnology to solve DoD-specific problems. Current approaches towards these aims tend to rely on introducing novel organisms (e.g., probiotics), engineering microbes and microbiomes, and leveraging unique capabilities of isolated microbes and microbiomes.

Although, DoD research and development communities focused on human health and performance tend to be separated from those focused on the environmental sciences, microbiome research transcends individual institutes and research missions. Sessions focused on emerging tools, enabling techniques, and product transition reinforced the need for inter-disciplinary teams, and DoD-wide communication and resource-sharing. Further, to fully leverage the power of microbiomes for military-specific biotechnology and other applications, a key foundational step will be continued DoD investment in fundamental research on human and environmental microbiomes, and in developing and enhancing novel tools for studying and engineering these communities. Of importance is that new findings are shared and new collaborations are developed to propel discoveries to useful biotechnology. Ideally, those opportunities will include mechanisms for DoD partnership with academia and industry in order to apply diverse expertise and capabilities to solving critical military-specific issues that may not otherwise be addressed. DoD agency funding mechanisms including, but not limited to, Multidisciplinary University Research Initiatives, Small Business Technology Transfer/Innovation Research programs, Laboratory University Collaboration Initiatives, Defense University Research Instrumentation Programs have proven fruitftul for establishing collaborations between government and private sectors to collectively elucidate insights into environment-host-microbiota interactions that have been incorporated into DoD research efforts. Leveraging those and similar funding mechanisms, sharing capabilities and expertise, and, perhaps most importantly, continually communicating the latest advances to avoid redundancy and maximize resources will remain critical to realizing the full impact of microbiome-based solutions to advance DoD interests.

## Data Availability

Not applicable.

## Abbreviations

DoD: US Department of Defense
MoBE: Microbiome of the built environment
TSMC: Tri-Service Microbiome Consortium
